# Machine learning of honey bee olfactory behavior identifies repellent odorants in free-flying bees in the field

**DOI:** 10.7554/eLife.104831

**Published:** 2026-09-23

**Authors:** Joel Kowalewski, Barbara F Baer-Imhoof, Tom Guda, Matthew Luy, Payton DePalma, Boris Baer, Anandasankar Ray

**Affiliations:** 1 Department of Molecular, Cell and Systems Biology, University of California, Riverside Riverside United States; 2 Department of Entomology, Centre for Integrative Bee Research (CIBER), University of California, Riverside Riverside United States; 3 Interdepartmental Neuroscience Program, University of California, Riverside Riverside United States; New York University United States; New York University United States

**Keywords:** honey bee, olfaction, repellent, behavior, machine learning, *Apis mellifera*, *D. melanogaster*, Other

## Abstract

Preventing beneficial insects like honey bees (*Apis mellifera*) from contacting pesticides on crops using odorants could counter current pollinator declines. However, the discovery of behaviorally aversive odorants is impeded by the complexity of the honey bee olfactory system where >170 olfactory receptors detect volatiles and generate valence. To solve this systems-level challenge, we generated a machine-learning model to predict aversive valence from chemical structure using published olfactory behavior data in honey bees. We refine the predictive model by generating species-level behavioral data for honey bees and *Drosophila* on an initial set of novel predicted repellents. The improved second computational model was then used to screen a chemical space of >50 million compounds and identify >130 repellent candidates. Behavioral validation using honey bees in the laboratory shows a high predictive success. Additional testing of the top seven candidates using freely foraging honey bees in a field assay confirmed strong repellency, thus predicting a high probability to repel foraging bees from pesticide-treated crops. Machine learning, with iterative testing and modeling, therefore provides a powerful approach for rational discovery of aversive volatiles for control of insects for which limited data is available.

## Introduction

Honey bees play a critical role as pollinators for nearly a third of food crops (Eilers et al., 2011). However, over the last decade, beekeepers reported increasingly unsustainable losses (>60%) of their managed hives every year. Several factors have been identified contributing to these declines, among them parasites such as *Varroa* mites, climate change, and pesticides (Feazel-Orr et al., 2016; Grassl et al., 2018; Wu et al., 2011).

When honey bee workers forage in pesticide-treated fields, they bring back pesticide residues into their hive, which negatively impacts the survival of their colony, endangering the pollination of high-value crops, even at sublethal dosages. We propose a novel solution for this problem: identifying honey bee repellents that can be added to pesticides, thus reducing their contact with treated crops. Honey bees have a powerful sense of smell and use their antennae to detect numerous volatile chemicals, which they sense using a sophisticated olfactory system of >170 odorant receptor genes and 21 IR genes in the genome that encode for odor-gated ion channels (Robertson and Wanner, 2006; Kelber et al., 2006). However, the complexity of the insect olfactory system makes it a significant challenge to experimentally identify repellent odorants. In fact, in the 20 years since the odorant receptor families in insects have been identified, not a single new repellent odorant was registered for use in a new insect repellent product by the U.S. Environmental Protection Agency (EPA).

**Figure 1.**
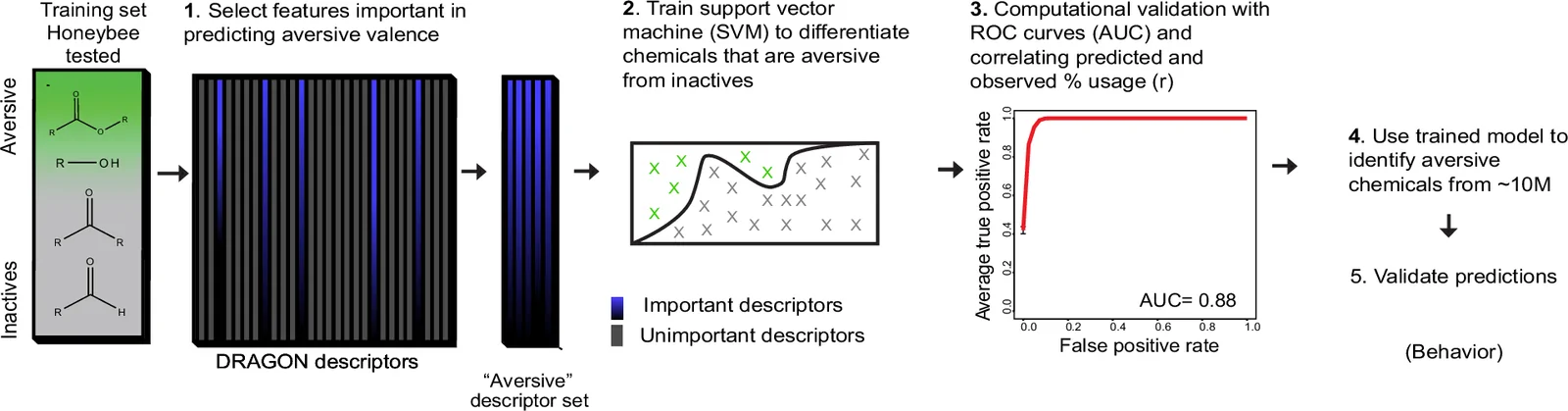
A chemical informatics method to predict honey bee repellence. Overview of the steps for the machine learning-based cheminformatics pipeline used to model honey bee repellence from the 3D structure of a training set of known repellents, and subsequent in silico screening for novel repellents from a large chemical space.

Recently, we used machine learning (ML) to successfully create structure-based models to predict mosquito repellents from chemical structure (Kowalewski et al., 2024) and for human olfactory behavior, allowing us to predict ~140 odor characters with great success (Kowalewski and Ray, 2020; Kowalewski et al., 2021). While a large empirical dataset of repellent odorants is available for mosquitoes, comparable data for honey bees are much more limited (Kowalewski et al., 2024). Here, we used a related ML approach, scanning 3D molecular structures of odorants in silico, to predict their potential for repelling honey bees (Figure 1). We overcame the challenges of a small set of training odorants by performing a second round of modeling using data from an initial round of testing to improve the predictive ability .

To do so, we first reviewed the existing literature on honey bee olfactory behavior and assembled a training set of volatile chemicals, including a subset with a negative behavioral valence (Melksham et al., 1988; Woodrow et al., 1965; Atkins et al., 1975; Milet-Pinheiro et al., 2013; Jones, 1952). We generated energy-minimized 3D structures for each chemical and calculated 5290 physicochemical features: initially for 184 training chemicals and later for 203 chemicals. From the initial training set, we identified 45 features that were considered important for predicting aversive valence (Supplementary file 1). We then evaluated a collection of ML algorithms, including regularized random forest, gradient-boosted decision trees, and a nonlinear support vector machine (e.g. incorporating the radial basis function kernel) with these features to predict repellency of the test chemicals. To cross-validate each algorithm’s performance, we divided the training data into multiple smaller training datasets and test datasets and calculated average predictive success, graphically depicted as the area under the receiver operating characteristic curve (ROC AUC) in our model. Our computational validation (avg AUC = 0.88) indicated that our model was able to distinguish volatiles with aversive valence from non-repellents (Figure 1).

Using this model, we subsequently evaluated a library of ~45 million small molecules from the MolPort database. The top predicted repellents (~7% of hits with computed repellency scores ≥ 0.9) represented a structurally diverse group of 139 chemicals, of which some showed similarities to the known repellents in the training set. The notion that ML can predict the behavioral valence of small molecules toward honey bees from just 3D chemical structure would be innovative and inform follow-up behavioral validation.

**Figure 2.**
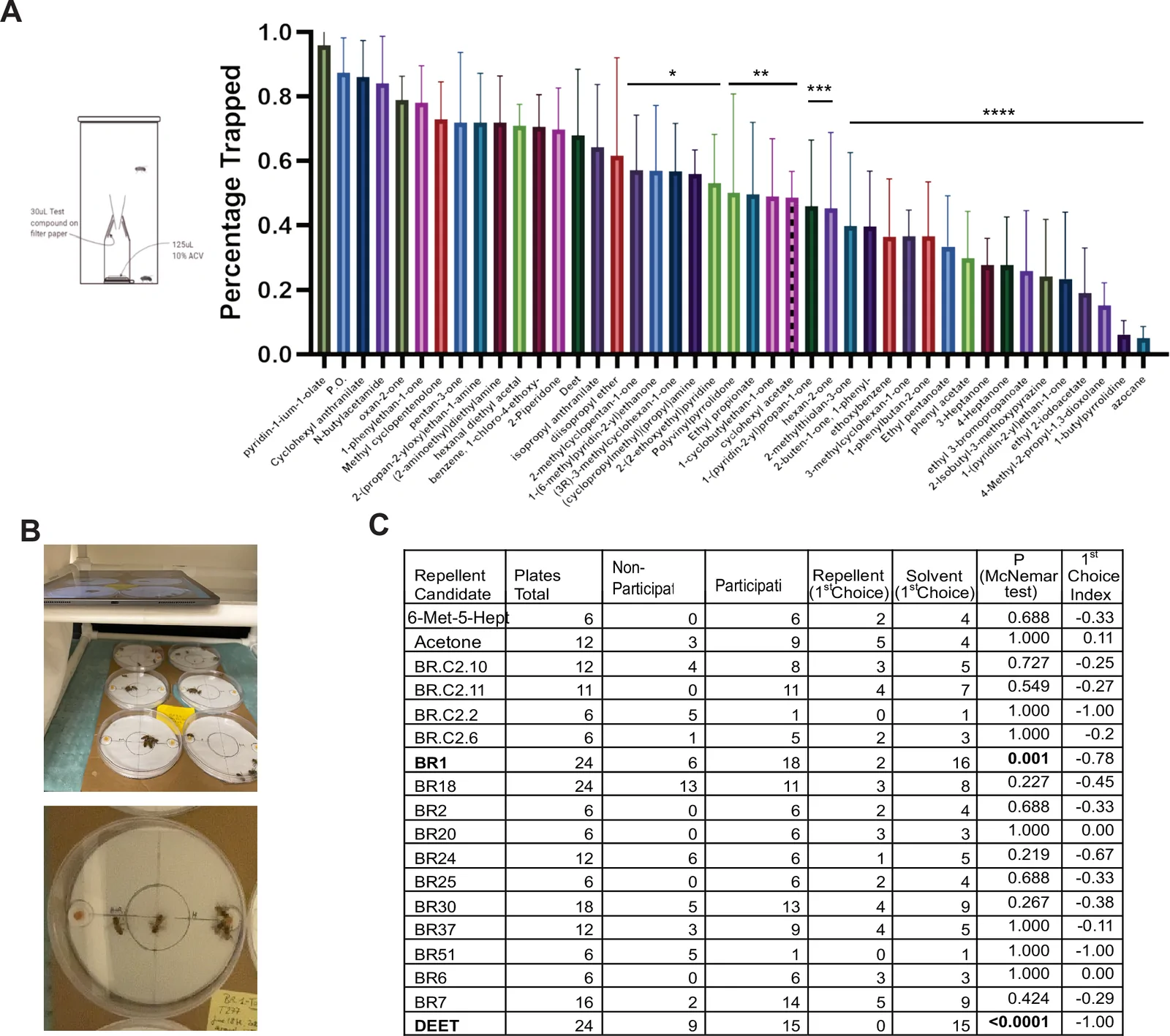
Predicted honey bee repellents. (**A**) Testing chamber containing a one-choice trap to determine whether an odorant will repel fruit flies, and the mean percentage of fruit flies caught in a trap treated with predicted repellent odorants (10% vol/vol in paraffin oil) and baited with 10% apple cider vinegar. N=5–10 trials (~20 flies/trial). Error bars = s.e.m. *p<0.05, **p<0.005, ***p<0.001, ****p<0.0001. (**B**) Photograph of the two-choice Petri dish arenas used to test the behavior of honey bees to repellent candidates individually. (**C**) Table with preference indexes in the two-choice assay for the first choices of honey bee workers offered two containers of honey water (50 μl of 50%) placed on filter paper disks treated with a test chemical or treated with solvent alone. The index was calculated for each compound as (total number of repellent choices minus total number of solvent choices) divided by the sum of all tests. We used McNemar tests for pairwise comparisons. Figure 2—source data 1.Data for *D. melanogaster* trap assays.

Because our training set included a few previously known repellents for other insects as well, such as benzaldehyde and geraniol, we anticipated that the predictions likely included broad-spectrum insect repellents alongside honey bee-selective ones. If repellents were to be mixed with insecticides to repel bees, it might be desirable not to repel pest insects. To evaluate predicted compounds for broad-spectrum insect repellent activity, we performed behavior assays using another insect, the fruit fly *Drosophila melanogaster* (Figure 2A). We randomly selected a set of compounds from the predictions and used a trap-based assay to quantify fly aversion. Several compounds showed low levels of repellency, with a <50% reduction in numbers of flies entering traps relative to the solvent control (Figure 2A). Subsequently, we only used those compounds for further tests in honey bees.

We modified a honey bee repellent testing assay in Petri dishes in the lab to test the 15 candidate compounds (Larson and Anderson, 2017). We placed honey water (50 μl) filled microfuge lids onto treated filter paper disks on two sides of a Petri dish and placed ~5 hungry honey bee workers in the center. From video recordings, we evaluated whether the first bee navigated to a honey pot on the repellent treated or the control solvent side (Figure 2B). For many of the tested compounds, the bees preferred to visit the honey water pots on the control side versus the repellent side (Figure 2C).

**Figure 3.**
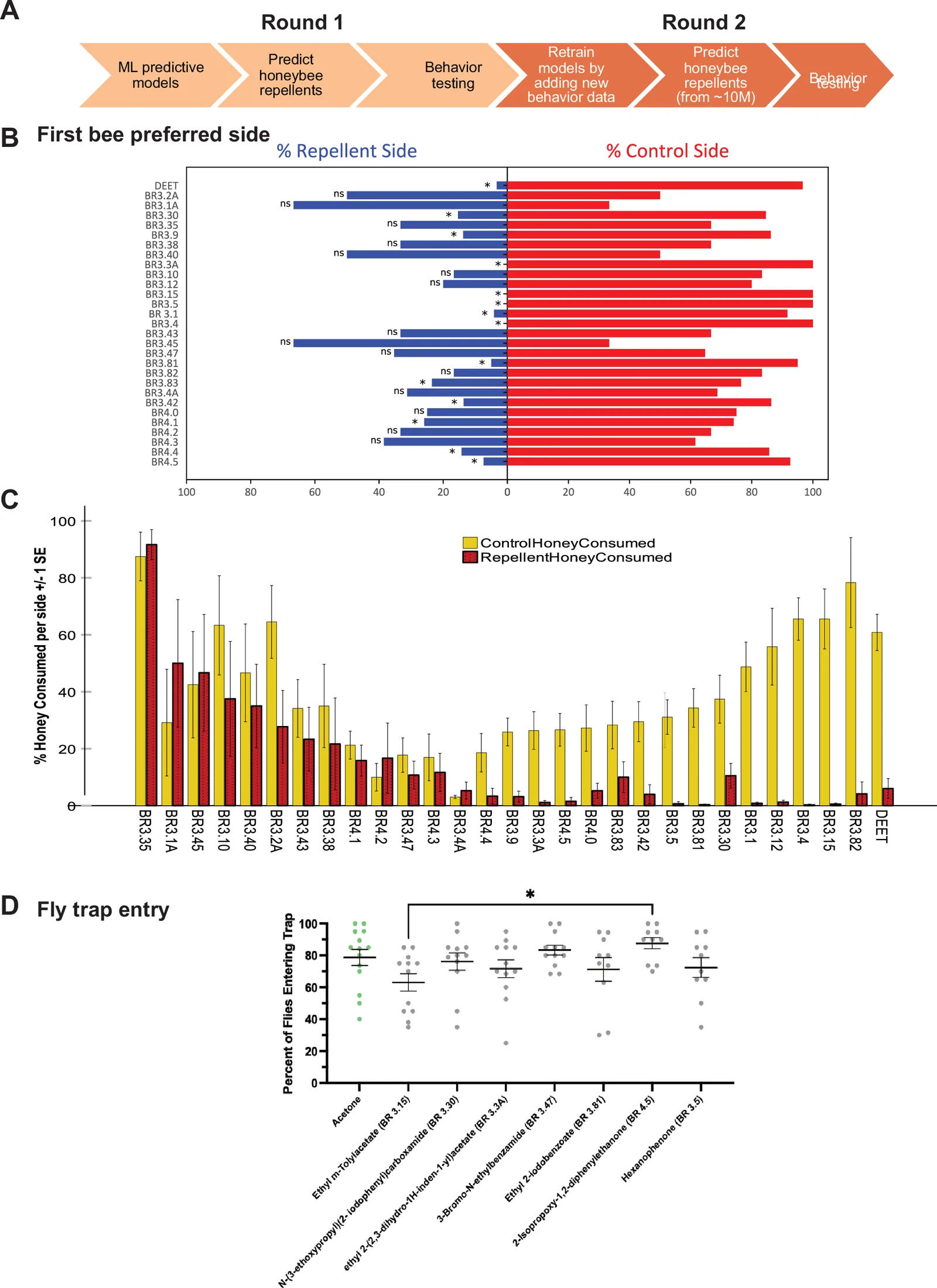
Reiterative training of machine learning (ML) predictive models and testing predicted honey bee repellents. (**A**) Schematic pipeline for the overall computational-behavior hybrid pipeline showing the two rounds. (**B**) Mean preference index of honey bees in making the first choice to move to the repellent treated side in a two-choice plate assay. 70 μl of pure honey was used in the two containers to attract the bees. (**C**) Average consumption of honey from containers placed on each side in the two-choice plate assays (N=460 plates). The index is calculated for each compound as which honey pot has greater consumption (total number of repellent side with greater consumption minus total number of solvent side with greater consumption) divided by the sum of all choices. (**D**) Mean percentage of fruit flies caught in a trap treated with predicted repellent odorants at application rates to test in field tests (0.1 mg/cm^2^) and baited with 10% apple cider vinegar. N=10–14 trials (~20 flies/trial). Error bars = s.e.m. Figure 3—source data 1.Fly trap entry data.

The initial training dataset used for ML was limited in size and lacked high-quality quantitative values for structurally related compounds. The predictive models can be improved substantially by adding new compounds with quantitative data that are structurally related to the training set. We therefore developed an iterative pipeline where the previous round of behavioral validation data is added to the training data and used to rebuild a more refined training set (Figure 3A). Specifically, we added the quantitative data from the 15 newly tested compounds to the training set. The 3D structures were analyzed, and a physicochemical feature set was selected as before (Supplementary file 1). As expected, the computational validation results based on the AUC values show an improvement. We next screened an ~10 million compound library in silico with the updated repellent predictive ML model and identified a wide array of natural and synthetic honey bee repellent candidates (Figure 3A). Because the rate of evaporation may affect how long a repellent will be effective in the field, we estimated the vapor pressure for each candidate. To do this, we used ML on a newly created training dataset of 1931 compounds with known vapor pressures.

**Figure 3—figure supplement 1.**
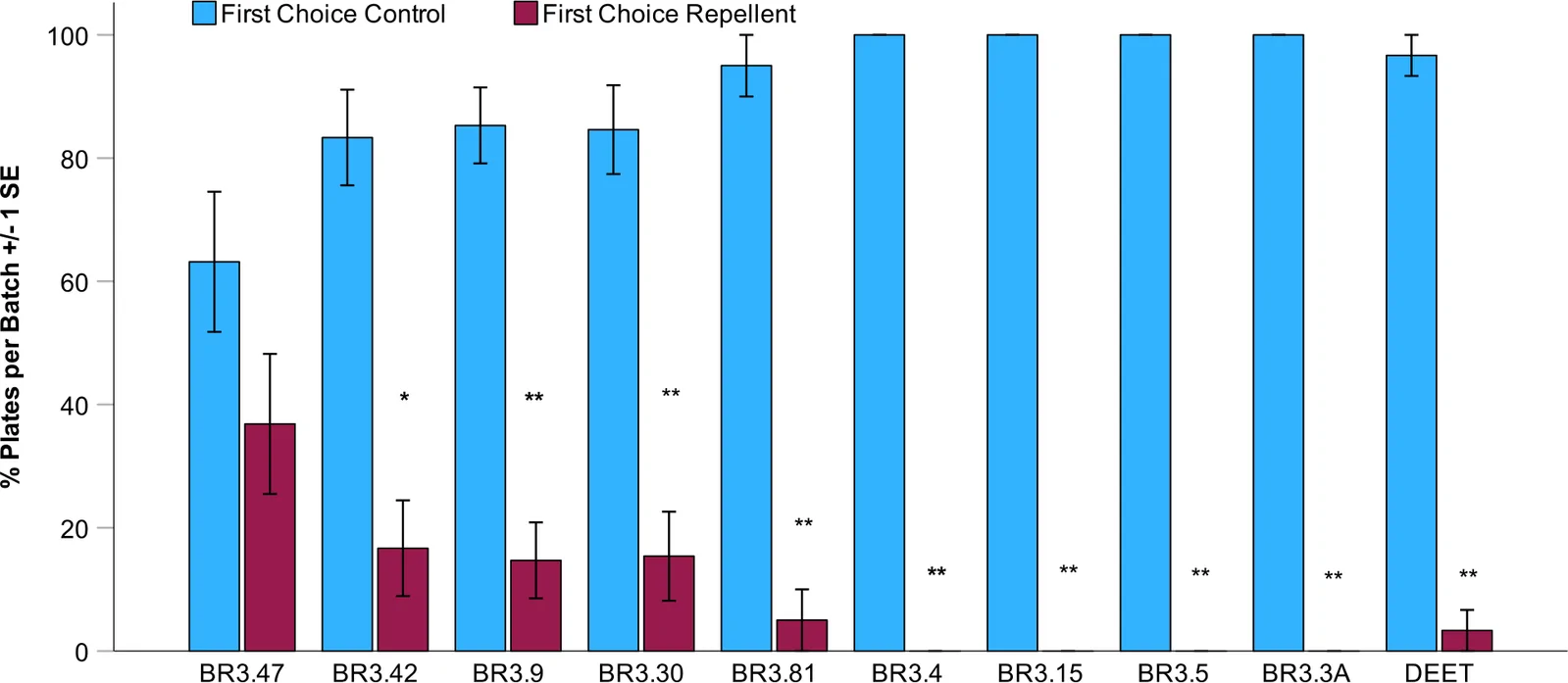
Average percentage of two-choice plate assays in which honey bees made their first choice to either move to the repellent treated side or solvent treated side across different honey bee colonies, N=3-6 colonies (~6 plates/colony). Error bars = 1 s.e.m., *p<0.05, **p<0.001.

To validate the second round of predictions and identify compounds with the strongest behavioral responses in honey bees, we selected 28 molecules with high predicted repellency values that fell into one of three vapor pressure (VP) categories: very low VP solids, very low VP liquids (10^–4^–10^–3^ mmHg at 25°C), and low VP liquids (10^–2^ mmHg at 25°C). We tested the compounds on honey bee behavior using the two-choice arena as before but used pure honey (70 μl) instead of honey water. Using honey increased the bees’ participation from 68% in round 1 to 81% in round two, and the increased volume allowed us to evaluate honey consumption on each side of the arena after 1 hr. Most of the predicted repellent compounds showed strong repellency (Figure 3B). The repellent effects were conserved across three or more different honey bee colonies when tested in batches, demonstrating a robust and reproducible effect (Figure 3—figure supplement 1). For 15 of the 28 test compounds, we found that the bees drank significantly less honey on the treatment side compared to the control side as well (Figure 3C, Supplementary file 2).

We next tested seven compounds that showed the strongest repellency in honey bees on *D. melanogaster* using the 24 hr trap assay as before, at a concentration relevant for field applications as done later (~0.1 mg/cm^2^). The flies showed no behavioral aversion to the candidate repellent chemicals, compared to controls (Figure 3D). This suggests that the new honey bee repellents were not aversive to other insects like fruit flies.

**Figure 4.**
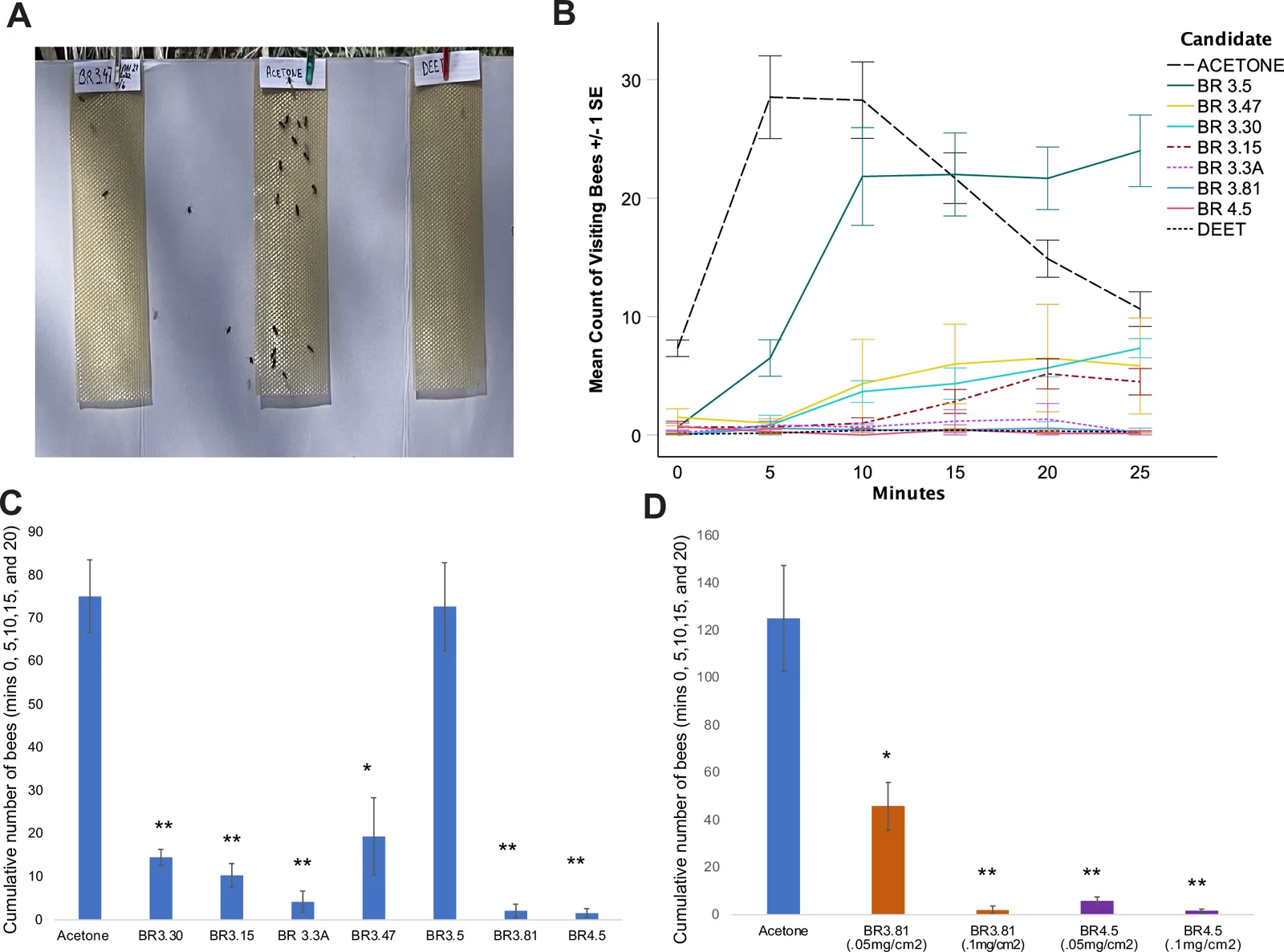
Field testing of top repellents using robbing assays. (**A**) Representative photo of a robbing assay. (**B**) Time course of the mean numbers of foraging honey bees visiting for each indicated compound tested (0.1 mg/cm^2^). DEET was used as a positive control and acetone as a negative control in each assay. N=6–18. Error bars = s.e.m. (**C**) Mean number of honey bees for each compound summed over the time points shown in B. *p<0.05, **p<0.005, ***p<0.001. We used Kruskal-Wallis tests for pairwise comparisons. (**D**) Mean number of honey bees for each compound tested at the regular dose (0.1 mg/cm^2^) and half dose (0.05 mg/cm^2^), summed over the time points over the duration of the experiment as above. N=4, error bars = s.e.m. Figure 4—source data 1.Data on honey bee robbing assays.

Next, we tested the seven repellents on freely flying foraging honey bees in a field assay. Honey bees are readily attracted to wax honeycomb foundations that have been sprayed with sugar water. We sprayed sugar water on three equally spaced wax foundations placed near hives and treated them with either (Atkins et al., 1975) solvent (acetone) (Dively et al., 2015), a candidate honey bee repellent (rate of ~0.1 mg/cm^2^), or (Eilers et al., 2011) a positive control (DEET) at the same rate (Figure 4A). The honey bees flew to the wax foundations and were offered a choice between the different treatments. We filmed the assays and counted the bees on each foundation in 5 min increments. Each of the seven compounds showed repellency to honey bees, with three of them showing aversion up until the end of the assay. We ended the assay counts when the honey bees stopped being attracted to the untreated controls (Figure 4A, B, and C). Kruskal-Wallis tests revealed significantly lower numbers of bees on treated wax foundations compared to controls (Figure 4C). In addition, we tested the two top-scoring compounds at half their original concentration (0.05 mg/cm^2^) and still observed significant repellency (Figure 4D).

Taken together, our results from the lab and field assays demonstrate that a computational approach can successfully model honey bee aversive valence from chemical structure alone and can be used to efficiently screen millions of chemicals to find strong repellents.

## Discussion

Most odorants reaching a bee’s antennae activate or inhibit several of the >160 olfactory receptors at the same time. Each odorant-receptor interaction elicits a specific neuronal activity in the bee brain, consequently allowing for a limited number of receptors to encode a practically infinite number of olfactory combinations, with >160 OR and 21 IR genes in the genome (reviewed in Paoli and Galizia, 2021). Even though honey bees are a well-studied model system for olfactory coding and learning (Paoli and Galizia, 2021; Sandoz, 2011), we still know little about the pathways between olfactory stimulus evaluation and decision-making (Paoli and Galizia, 2021). The olfactory system therefore poses an extreme challenge to modeling. In spite of what is already known about the honey bee olfactory system, it would take several years and significant funding before generating a near-complete knowledge of the olfactory system that allows us to do predictive modeling of valence. The 3D structure-based computational approach fills a very important void in identifying novel volatiles triggering a specific olfactory behavioral response in honey bees. This ML pipeline can accelerate research into insect repellency and its physicochemical basis. The overall species-selective approach we have presented will help identify additional novel repellent chemicals for honey bees, as well as inspire an exploration of the vastness of chemical space for use in repellent strategies for other animals.

Pesticides have been identified as a significant contributor to the global decline in honey bee populations (Goulson et al., 2015; Potts et al., 2010). Numerous studies have demonstrated the detrimental effects of various pesticides alone or in combination with other environmental stressors on honey bee health, including neonicotinoids, organophosphates, pyrethroids (Whitehorn et al., 2012; Rundlöf et al., 2015), and bifenazate (Wang et al., 2020). These chemicals can cause acute toxicity, leading to immediate bee mortality, as well as sublethal effects that impair bee behavior, navigation, immune function, and population growth (Wang et al., 2020; Henry et al., 2012). Chronic exposure to pesticides, such as imidacloprid, has been linked to reduced colony growth, queen failure, and increased susceptibility to pathogens and parasites (Dively et al., 2015). The widespread use of pesticides in agriculture has raised concerns about the sustainability of honey bee populations and the potential impacts on pollination services and food security (Potts et al., 2010).

Recent research has explored the potential of using bee repellent odors to mitigate the harmful effects of pesticides on honey bees. The concept involves applying non-toxic, natural compounds that emit odors that are unappealing to bees, thereby deterring them from foraging in treated areas during pesticide application. By reducing bee exposure to pesticides, this approach aims to minimize the direct and indirect impacts on bee health. Studies have identified several promising bee repellent compounds, such as 2-heptanone and essential oils from plants like lavender and thyme, but their low efficacy does not make them practical in the agricultural setting (Matos et al., 2021; Rieth et al., 1986). Our field behavior assays strongly suggest that the use of bee repellent odors can significantly reduce bee activity in treated areas, potentially lowering the risk of pesticide exposure. This study also provides a foundation for further research to optimize the effectiveness of bee repellent odors, assess their long-term impacts on bee behavior and colony health, and develop practical application strategies for integration into pest management practices. Moreover, our approach for a rational and high-throughput discovery of bee repellents can be extended to any other species of interest, thus providing a very powerful and affordable computational tool for chemical ecologists.

## Materials and methods

### ML repellency

Each chemical structure tested for attraction/repellency on bees was prepared for ML using RDKit in Python. The 2D structures were first embedded in 3D coordinate space; the 3D geometries were then optimized using the Merck molecular force field (MMFF94). For each optimized 3D structure, AlvaDesc computed 5290 diverse 2D and 3D chemical features, describing large-scale properties, such as molecular shape, that affect sensory receptor docking in addition to forces describing potentially more abstract interactions leading to graded activation or inhibition of the sensory receptors of the bee.

Bee behavioral data, presented as a preference score (5=max repellency), was converted into a binary label, repel/inactive, using >1 as the cutoff between repellent and neutral or attractant behaviors. In other circumstances, bee behavior to compounds was reported without a repellency score. For attractive compounds, an inactive label was assigned based on previous data (Woodrow et al., 1965; Atkins et al., 1975; Milet-Pinheiro et al., 2013; Jones, 1952; Melksham et al., 1988; Liao et al., 2017). The data were subsequently split into training and testing partitions (90:10), and any further processing was done exclusively on the training partition.

The large chemical feature matrix was next reduced to a subset of features that optimally classified compounds according to their repellent activity on bees. This involved using the recursive feature elimination (RFE) algorithm alongside a support vector machine (SVM) with a radial basis function (RBF) kernel to evaluate the feature importance over 250 partitions of the training data (e.g. 10-fold cross-validation, repeated 25 times). An intermediate subset >100 features was next passed to a series of algorithms (e.g. random forest and boosted decision trees) to re-rank the features, selecting the final, consensus set.

Top features were evaluated across a diverse set of algorithms, including SVMs, decision trees, and neural networks (multilayer perceptron), and the cross-validated classification metrics suggested high classification success rates (AUC, sensitivity, and specificity) when aggregating the predictions of three algorithms: SVM with an RBF kernel, a gradient boosting machine (built with decision trees), random forest with regularization. Final evaluation was done on the test set. Trained models predicted 60,000 metabolites and other naturally sourced compounds. The MolPort database, which contains 47 million compounds in total, was filtered to a smaller subset for repellency predictions, based on commercial availability and structural similarity to known bee repellents in the training set. After the first round of experimental validation tests with bees, the models were retrained. Additional prospective repellent compounds were scored using the updated models.

### ML vapor pressure

Training and testing data were obtained from EPI suites, EPA. Methods for fitting these models are as outlined for bee repellency; namely, chemical features were computed using AlvaDesc, followed by processing the feature values and identifying a small predictive subset through the RFE algorithm. To compare the vapor pressure model predictions with respect to different ML methods, as well as EPI suite, data were split according to the train/test partitions defined in a previous study using the vapor pressure data for ML (Zang et al., 2017).

### Fly trap assay

*D. melanogaster* stocks (RRID:BDSC_1) maintained in the lab were grown in standard media bottles. All chemicals were purchased at highest purity through MolPort. Flies of a similar age (4–6 days) were collected in groups of 20 (10 females+10 males) and wet-starved for 24 hr in starvation vials. Testing chambers (Dram vials with centrifuge tube traps containing lure [10% apple cider vinegar] plus the test compound of desired dilutions) were assembled, and flies quickly transferred to them by gentle tapping. The assays were left on the bench, and data on the number of flies entering traps recorded after 18 hr.

### Two-choice honey bee plate assays

Honey bee (*Apis mellifera*) workers for our experiments were bred in apiaries at the University of California Riverside, between August 2021 and April 2022. To age them, we placed a frame of capped worker brood at the red-eye stage into an observation frame in a humidified incubator at 34°C. After 2–3 days, we collected groups of 80 newly hatched workers into specially 3D-printed experimental cages (12×10×7.5 cm^3^), where we kept them until they reached an age of 13–19 days, corresponding to foraging age. The bees received sugar water (50%) and water ad libitum, as well as a paste of protein powder (https://megabee.com/) mixed with 50% sugar water every second day until day 8.

The behavior arenas consisted of plastic Petri dishes with a diameter of 15 cm with two filter paper circles on each side (1 cm diameter) treated with 20 μl of solvent (acetone evaporated for 30 min) on one side and 20 μl of test compound (20 μl, 5% in acetone, evaporated for 30 min) on the opposite side of the arena. To stimulate bees to participate, 50 μl of 50% honey water in cut caps of PCR tubes was carefully taped to the center of each of the two treated papers (Figure 2). For round two (Figure 3), 70 μl of pure (slightly warmed) honey was used in each cap (the honey surface formed an upward meniscus). The night before the experiment, the bees were water-starved, then cooled down at 4°C for approximately 20 min before placing five bees into the middle of the arena. The spacing of the filter papers was kept consistent by taping a template (round piece of paper) to the bottom of each Petri dish and taping the filter papers on top of the correct spot onto the template. An Apple iPad or iPhone was used to film six arenas at once for 1 hr in a custom-built PVC tube cage covered with a cotton sheet to create a uniform visual surrounding. To wake up the bees, the choice arenas were placed onto a heating pad (20''×24'', Model No PEHPWIDE-SG, Pure Enrichment, purenrichment.com, set to level 1).

Videos were analyzed offline. For each plate, the side the first bee chose to drink honey from first was recorded (first choice). An additional semiquantitative metric was noted for Figure 3: an estimate of the percentage of honey consumed in each container 95% (meniscus gone, only), 75%, 50%, 25%, or 0%. Plates in which the bees had not consumed any honey from either side (non-participating plates) were excluded from further evaluations. For each batch of six plates, workers from the same colony were used. For repellent candidates with the most promise, bees from at least three different colonies were tested (at least six participating plates each) (Supplementary file 2).

### Robbing assays in the field

For this assay, we elicited the robbing behavior of honey bees, which means that they steal unguarded honey or honey guarded by weaker colonies (Free, 1954). To do so, we sprayed standardized wax foundations (44×25.5 cm^2^) with 50 puffs of 50% honey diluted in water as an attractant. Chemicals were sprayed on top at 0.1 mg/cm^2^ (15 puffs of 5% in acetone). Three foundations, solvent, DEET, and test compound, were affixed on a standing poster board (92×122 cm^2^) placed on a table 5 m in front of the first row of 40 hives and filmed for robbing behavior by the bees for 30 min, using an iPad or an iPhone. Prior to the start of the day’s experiments, three honey frames and honey lures were placed on a table for ~15–30 min to trigger robbing behavior of bees from nearby colonies and then removed before trials. The position of the three wax foundations to each other was randomly shifted. After 25 min, the positive control (sugar-water-sprayed wax foundations) was no longer attractive to honey bees, so the analysis was restricted to this time point. We repeated each assay a minimum of six times. Moreover, we repeated the assays with the two most promising candidates at half the first concentration (~0.05 mg/cm^2^) four times each.

### Statistics

To compare the proportion of plates per test batch in which the bees chose to drink honey from the control side versus the treated sides, we used non-parametric, pairwise McNemar tests, assuming the null hypothesis of an equal distribution between choices or 50/50. The non-parametric, pairwise Wilcoxon signed-rank tests were used to compare the percentage of honey consumption between treatments. Plates in which bees did not drink any honey were removed (non-participating plates). For the assays on *Drosophila* behavior, we performed ANOVA followed by Tukey’s multiple comparison tests to compare the number of flies entering the trap between treatments and control. For the robbing assays, we summed up the counts of visiting bees on each wax foundation at 5, 10, 15, and 20 min, then used non-parametric Kruskal-Wallis tests for pairwise comparisons between each of the three treatments. We adjusted the p-values for multiple testing with Bonferroni corrections.

### Resource availability

#### Lead contact

Anandasankar Ray, anand.ray@ucr.edu.

#### Materials availability

No new materials were generated in this study.

## Data Availability

Data used in the analyses is publicly available from the references cited, and the data generated in this manuscript are supplied within the figures, Supplementary Files, and Source Data Tables. The code developed for this study is available from the corresponding author for non-commercial use upon request. Use of the code may be subject to a standard Material Transfer Agreement (MTA) or specialized licensing agreement through University of California Riverside's Technology Transfer Office, as patent applications related to this invention are currently pending.

## Additional files

Supplementary file 1. Optimized DRAGON physicochemical descriptor set from iterative training in Figure 3A used to predict bee repellent compounds.

Supplementary file 2. Repellency testing in the two-choice plate assay from the second iteratively trained round of predictions.

Supplementary file 3. Training chemicals used for the ML. (a) Initial data for Round 1 machine learning. (b) Data added for Round 2 after testing for bee repellency.

MDAR checklist

## References

[bib1] Atkins EL, Macdonald RL, McGovern TP, Beroza M, Greywood-Hale EA (1975). Repellent additives to reduce pesticide hazards to honeybees: laboratory testing. Journal of Apicultural Research.

[bib2] Dively GP, Embrey MS, Kamel A, Hawthorne DJ, Pettis JS (2015). Assessment of chronic sublethal effects of imidacloprid on honey bee colony health. PLOS ONE.

[bib3] Eilers EJ, Kremen C, Smith Greenleaf S, Garber AK, Klein AM (2011). Contribution of pollinator-mediated crops to nutrients in the human food supply. PLOS ONE.

[bib4] Feazel-Orr HK, Catalfamo KM, Brewster CC, Fell RD, Anderson TD, Traver BE (2016). Effects of pesticide treatments on nutrient levels in worker honey bees (*Apis mellifera*). Insects.

[bib5] Free JB (1954). The behaviour of robber honeybees. Behaviour.

[bib6] Goulson D, Nicholls E, Botías C, Rotheray EL (2015). Bee declines driven by combined stress from parasites, pesticides, and lack of flowers. Science.

[bib7] Grassl J, Holt S, Cremen N, Peso M, Hahne D, Baer B (2018). Synergistic effects of pathogen and pesticide exposure on honey bee (*Apis mellifera*) survival and immunity. Journal of Invertebrate Pathology.

[bib8] Henry M, Béguin M, Requier F, Rollin O, Odoux JF, Aupinel P, Aptel J, Tchamitchian S, Decourtye A (2012). A common pesticide decreases foraging success and survival in honey bees. Science.

[bib9] Jones GDG (1952). The responses of the honey-bee to repellent chemicals. Journal of Experimental Biology.

[bib10] Kelber C, Rössler W, Kleineidam CJ (2006). Multiple olfactory receptor neurons and their axonal projections in the antennal lobe of the honeybee *Apis mellifera*. The Journal of Comparative Neurology.

[bib11] Kowalewski J, Ray A (2020). Predicting human olfactory perception from activities of odorant receptors. iScience.

[bib12] Kowalewski J, Huynh B, Ray A (2021). A system-wide understanding of the human olfactory percept chemical space. Chemical Senses.

[bib13] Kowalewski J, Boyle SM, Arvidson R, Ejercito J, Ray A (2024). Machine learning based modelling of human and insect olfaction screens millions of compounds to identify pleasant smelling insect repellents. eLife.

[bib14] Larson NR, Anderson TD (2017). Video tracking protocol to screen deterrent chemistries for honey bees. Journal of Visualized Experiments.

[bib15] Liao LH, Wu WY, Berenbaum MR (2017). Behavioral responses of honey bees (*Apis mellifera*) to natural and synthetic xenobiotics in food. Scientific Reports.

[bib16] Matos WB, Santos ACC, Lima APS, Santana EDR, Silva JE, Blank AF, Araújo APA, Bacci L (2021). Potential source of ecofriendly insecticides: essential oil induces avoidance and cause lower impairment on the activity of a stingless bee than organosynthetic insecticides, in laboratory. Ecotoxicology and Environmental Safety.

[bib17] Melksham KJ, Jacobsen N, Rhodes J (1988). Compounds which affect the behaviour of the honeybee, *Apis mellifera* L.: a review. Bee World.

[bib18] Milet-Pinheiro P, Ayasse M, Dobson HEM, Schlindwein C, Francke W, Dötterl S (2013). The chemical basis of host-plant recognition in a specialized bee pollinator. Journal of Chemical Ecology.

[bib19] Paoli M, Galizia GC (2021). Olfactory coding in honeybees. Cell and Tissue Research.

[bib20] Potts SG, Biesmeijer JC, Kremen C, Neumann P, Schweiger O, Kunin WE (2010). Global pollinator declines: trends, impacts and drivers. Trends in Ecology & Evolution.

[bib21] Rieth JP, Wilson WT, Levin MD (1986). Repelling honeybees from insecticide-treated flowers with 2-heptanone. Journal of Apicultural Research.

[bib22] Robertson HM, Wanner KW (2006). The chemoreceptor superfamily in the honey bee, *Apis mellifera*: expansion of the odorant, but not gustatory, receptor family. Genome Research.

[bib23] Rundlöf M, Andersson GKS, Bommarco R, Fries I, Hederström V, Herbertsson L, Jonsson O, Klatt BK, Pedersen TR, Yourstone J, Smith HG (2015). Seed coating with a neonicotinoid insecticide negatively affects wild bees. Nature.

[bib24] Sandoz JC (2011). Behavioral and neurophysiological study of olfactory perception and learning in honeybees. Frontiers in Systems Neuroscience.

[bib25] Wang Y, Zhang J, Sun Y, Yu J, Zheng Z, Li S, Cui Z, Hao J, Li G (2020). Effects of intermittent mixing mode on solid state anaerobic digestion of agricultural wastes. Chemosphere.

[bib26] Whitehorn PR, O’Connor S, Wackers FL, Goulson D (2012). Neonicotinoid pesticide reduces bumble bee colony growth and queen production. Science.

[bib27] Woodrow AW, Green N, Tucker H, Schonhorst MH, Hamilton KC (1965). Evaluation of chemicals as honey bee attractants and repellents1. Journal of Economic Entomology.

[bib28] Wu JY, Anelli CM, Sheppard WS (2011). Sub-lethal effects of pesticide residues in brood comb on worker honey bee (*Apis mellifera*) development and longevity. PLOS ONE.

[bib29] Zang Q, Mansouri K, Williams AJ, Judson RS, Allen DG, Casey WM, Kleinstreuer NC (2017). In silico prediction of physicochemical properties of environmental chemicals using molecular fingerprints and machine learning. Journal of Chemical Information and Modeling.

