# Supplementary material for: Machine learning of honey bee olfactory behavior identifies repellent odorants in free-flying bees in the field
Source: eLife. 2026 Sep 23;14:RP104831. doi: 10.7554/eLife.104831 (PMC13600694; doi:10.7554/eLife.104831)
Supplement: Supplementary file 2 [file elife-104831-supp1.docx]

Supplementary file 1. Optimized DRAGON physicochemical descriptor set from iterative training in Figure 3A used to predict bee repellent compounds.
