# Supplementary material for: Machine learning of honey bee olfactory behavior identifies repellent odorants in free-flying bees in the field
Source: eLife. 2026 Sep 23;14:RP104831. doi: 10.7554/eLife.104831 (PMC13600694; doi:10.7554/eLife.104831)
Supplement: Supplementary file 3 [file elife-104831-supp2.docx]

Supplementary file 2. Repellency testing in the 2-choice plate assay from second iteratively trained round of predictions.

| **#** | **Repellent Candidate** | **Batches Tested** | **Names of compounds** | **Colonies Tested** | **Plates Total** | **Not Participating** | **Participating** | **1^st^ Choice Repellent** | **1^st^ Choice Control** | **1^st^ Choice**  **Control vs Repellent Mc Namar P** | **Honey Consumption**  **Wilcoxon P** | **1^st^ Choice Preference**  **Index*** |
| --- | --- | --- | --- | --- | --- | --- | --- | --- | --- | --- | --- | --- |
| 1 | BR3.1 | 3 | phenyl 3-methoxypropanoate | 2 | 24 | 5 | 19 | 1 | 18 | < 0.0001 | <0.001 | -0.895 |
| 2 | BR3.10 | 1 | (1R,2R)-2-ethoxycyclohexan-1-ol | 1 | 6 | 0 | 6 | 1 | 5 | 0.219 | 0.344 | -0.667 |
| 3 | BR3.12 | 2 | 1-methyl-2,3-dihydro-1H-indole | 2 | 12 | 1 | 11 | 2 | 9 | 0.065 | 0.005 | -0.636 |
| 4 | BR3.15 | 3 | ethyl 2-(2-methylphenyl)acetate | 3 | 18 | 0 | 18 | 0 | 18 | < 0.0001 | <0.001 | -1.000 |
| 5 | BR3.1A | 1 | 2-Methyl-N-propylbenzamide | 1 | 6 | 3 | 3 | 2 | 1 | 1.000 | 0.180 | 0.333 |
| 6 | BR3.2A | 2 | 2-(3-Oxo-3-phenylpropyl)cyclopentanone | 2 | 11 | 0 | 11 | 4 | 7 | 0.549 | 0.127 | -0.273 |
| 7 | BR3.30 | 5 | N-(3-ethoxypropyl)-2-iodobenzamide | 3 | 29 | 3 | 26 | 4 | 22 | < 0.001 | <0.001 | -0.692 |
| 8 | BR3.35 | 1 | butyl 3-methylquinoline-4-carboxylate | 1 | 6 | 0 | 6 | 2 | 4 | 0.688 | 0.705 | -0.333 |
| 9 | BR3.38 | 1 | 3-bromo-N-propylbenzamide | 1 | 6 | 0 | 6 | 2 | 4 | 0.688 | 0.673 | -0.333 |
| 10 | BR3.3A | 6 | ethyl 2-(2,3-dihydro-1H-inden-1-yl)acetate | 5 | 36 | 15 | 21 | 0 | 21 | < 0.0001 | <0.001 | -1.000 |
| 11 | BR3.4 | 3 | 1-phenylhexan-3-one | 3 | 18 | 0 | 18 | 0 | 18 | < 0.0001 | <0.001 | -1.000 |
| 12 | BR3.40 | 2 | 2-butyl-2,3-dihydro-1H-indazol-3-one | 1 | 6 | 0 | 6 | 3 | 3 | 1.000 | 0.674 | 0.000 |
| 13 | BR3.42 | 5 | 1-{2-[(1E)-2-phenylethenyl]phenyl}ethan-1-one | 3 | 30 | 6 | 24 | 4 | 20 | 0.002 | 0.002 | -0.667 |
| 14 | BR3.43 | 2 | 1H-indol-3-yl acetate | 1 | 12 | 0 | 12 | 4 | 8 | 0.388 | 0.479 | -0.333 |
| 15 | BR3.45 | 1 | 1H-indol-4-yl acetate | 1 | 6 | 0 | 6 | 4 | 2 | 0.688 | 0.832 | 0.333 |
| 16 | BR3.47 | 4 | 3-bromo-N-ethylbenzamide | 3 | 20 | 1 | 19 | 7 | 12 | 0.359 | 0.271 | -0.263 |
| 17 | BR3.4A | 3 | ethyl naphthalene-1-carboxylate | 1 | 18 | 1 | 17 | 6 | 11 | 0.332 | 0.553 | -0.294 |
| 18 | BR3.5 | 4 | 1-phenylhexan-1-one | 4 | 36 | 15 | 21 | 0 | 21 | < 0.0001 | <0.001 | -1.000 |
| 19 | BR3.81 | 5 | ethyl 2-iodobenzoate | 4 | 30 | 10 | 20 | 1 | 19 | < 0.0001 | <0.001 | -0.900 |
| 20 | BR3.82 | 1 | methyl 4-bromo-2-formylbenzoate | 1 | 6 | 0 | 6 | 1 | 5 | 0.219 | 0.043 | -0.667 |
| 21 | BR3.83 | 3 | ethyl quinoline-5-carboxylate | 2 | 18 | 1 | 17 | 4 | 13 | 0.049 | 0.062 | -0.529 |
| 22 | BR3.9 | 8 | 1-(2,3-dihydro-1H-indol-1-yl)-3-ethoxypropan-1-one | 3 | 46 | 12 | 34 | 5 | 29 | < 0.001 | <0.001 | -0.706 |
| 23 | BR4.0 | 4 | N-Cyclohexylbenzamide | 2 | 22 | 4 | 18 | 5 | 13 | 0.096 | 0.037 | -0.444 |
| 24 | BR4.1 | 6 | Ethyl 2-(2-Cyanoanilino) acetate | 2 | 36 | 9 | 27 | 7 | 20 | 0.021 | 0.091 | -0.481 |
| 25 | BR4.2 | 1 | N-Phenyl-1-naphthylamine | 1 | 6 | 0 | 6 | 2 | 4 | 0.688 | 0.915 | -0.333 |
| 26 | BR4.3 | 3 | 1-(9H-Carbazol-9-yl)ethanone | 1 | 18 | 5 | 13 | 5 | 8 | 0.581 | 0.473 | -0.231 |
| 27 | BR4.4 | 3 | 3-(Pyridin-3-yl)-1-(pyridin-4-yl)prop-2-en-1-one | 1 | 18 | 3 | 15 | 3 | 12 | 0.035 | 0.025 | -0.600 |
| 28 | BR4.5 | 6 | 2-Isopropoxy-1,2-diphenylethanone | 2 | 36 | 12 | 24 | 2 | 22 | < 0.0001 | <0.001 | -0.833 |
| 29 | DEET | 5 | *N*,*N*-Diethyl-*m*-toluamide | 5 | 30 | 0 | 30 | 1 | 29 | < 0.001 | <0.001 | -0.933 |
