# Supplementary material for: Machine learning of honey bee olfactory behavior identifies repellent odorants in free-flying bees in the field
Source: eLife. 2026 Sep 23;14:RP104831. doi: 10.7554/eLife.104831 (PMC13600694; doi:10.7554/eLife.104831)
Supplement: Supplementary file 4 [file elife-104831-supp3.docx]

# **Supplementary file 3a**: Initial Data for Round 1 Machine Learning

| PubChem CID | Repellency |
| --- | --- |
| 102911 | Repel |
| 102981 | Repel |
| 1032 | Repel |
| 10402 | Inactive |
| 10931 | Inactive |
| 10976 | Repel |
| 111037 | Inactive |
| 1127 | Repel |
| 11357 | Repel |
| 11369 | Inactive |
| 11419 | Repel |
| 11579 | Inactive |
| 12291 | Repel |
| 12310947 | Inactive |
| 123233 | Inactive |
| 12403 | Repel |
| 12404 | Repel |
| 1254 | Inactive |
| 12587 | Inactive |
| 12901 | Inactive |
| 13002 | Inactive |
| 13008 | Repel |
| 13566 | Repel |
| 13629102 | Inactive |
| 136888 | Repel |
| 137414 | Repel |
| 13879 | Repel |
| 14240 | Inactive |
| 15105174 | Inactive |
| 15133 | Inactive |
| 1549026 | Inactive |
| 15624 | Repel |
| 16678 | Inactive |
| 170833 | Repel |
| 17100 | Inactive |
| 17355 | Repel |
| 176 | Repel |
| 180 | Inactive |
| 18954 | Inactive |
| 19079 | Inactive |
| 199294 | Inactive |
| 20745 | Inactive |
| 221558 | Repel |
| 225147 | Repel |
| 232703 | Inactive |
| 233220 | Inactive |
| 2345 | Repel |
| 235640 | Inactive |
| 240 | Repel |
| 244 | Repel |
| 24623 | Repel |
| 246831 | Repel |
| 246835 | Repel |
| 24847 | Inactive |
| 24981 | Repel |
| 24994 | Repel |
| 261 | Repel |
| 2879 | Inactive |
| 2969 | Inactive |
| 3004112 | Inactive |
| 3026 | Repel |
| 3102 | Repel |
| 311 | Inactive |
| 31229 | Repel |
| 31263 | Repel |
| 314154 | Inactive |
| 33191 | Repel |
| 3438 | Repel |
| 346056 | Inactive |
| 4004 | Inactive |
| 4115 | Inactive |
| 4133 | Repel |
| 428 | Repel |
| 4284 | Inactive |
| 442484 | Inactive |
| 454 | Repel |
| 522168 | Repel |
| 527 | Repel |
| 5282108 | Inactive |
| 5284503 | Inactive |
| 5312738 | Repel |
| 53167 | Repel |
| 532460 | Repel |
| 5354264 | Repel |
| 5354896 | Inactive |
| 5355850 | Inactive |
| 5356959 | Inactive |
| 5363140 | Repel |
| 5365800 | Repel |
| 5370101 | Repel |
| 5462719 | Inactive |
| 5634 | Inactive |
| 6028 | Inactive |
| 61004 | Inactive |
| 61067 | Repel |
| 61417 | Repel |
| 62350 | Inactive |
| 637511 | Repel |
| 637564 | Inactive |
| 637566 | Inactive |
| 638011 | Inactive |
| 638014 | Inactive |
| 64685 | Inactive |
| 6551 | Inactive |
| 6569 | Repel |
| 6590 | Repel |
| 66093 | Repel |
| 6673 | Inactive |
| 6781 | Repel |
| 6915 | Inactive |
| 6923 | Inactive |
| 69389 | Inactive |
| 69656 | Repel |
| 69702 | Inactive |
| 6985 | Repel |
| 6989 | Inactive |
| 70316 | Repel |
| 712 | Inactive |
| 7298 | Repel |
| 7410 | Repel |
| 7416 | Repel |
| 74741 | Repel |
| 7497 | Repel |
| 7507 | Inactive |
| 75200 | Inactive |
| 75454 | Inactive |
| 7554 | Repel |
| 75678 | Repel |
| 7590 | Repel |
| 75945 | Inactive |
| 7618 | Inactive |
| 76194 | Repel |
| 76388 | Inactive |
| 7677 | Inactive |
| 7678 | Repel |
| 7719 | Repel |
| 77321 | Inactive |
| 77323 | Inactive |
| 7746 | Repel |
| 7751 | Repel |
| 7757 | Repel |
| 7761 | Repel |
| 7762 | Repel |
| 7777 | Inactive |
| 77844 | Inactive |
| 7794 | Inactive |
| 78481 | Inactive |
| 7854 | Repel |
| 7912 | Repel |
| 7923 | Repel |
| 79306 | Repel |
| 7965 | Repel |
| 8007 | Repel |
| 8050 | Inactive |
| 8051 | Repel |
| 8094 | Inactive |
| 8096 | Inactive |
| 8102 | Repel |
| 81188 | Repel |
| 8148 | Repel |
| 8149 | Inactive |
| 8180 | Inactive |
| 8222 | Repel |
| 8257 | Inactive |
| 8268 | Inactive |
| 8294 | Inactive |
| 8332 | Inactive |
| 8471 | Repel |
| 8554 | Repel |
| 86079 | Inactive |
| 8635 | Inactive |
| 8797 | Repel |
| 8815 | Inactive |
| 8883 | Inactive |
| 8902 | Repel |
| 8916 | Repel |
| 95315 | Inactive |
| 95500 | Repel |
| 95633 | Repel |
| 969491 | Inactive |
| 98279 | Repel |
| 9862 | Repel |
| 996 | Repel |
| 999 | Inactive |

# **Supplementary file 3b**: Data added for Round 2 after testing for Bee Repellency

| PubChem CID | Repellency |
| --- | --- |
| 4284 | Repel |
| 6877 | Repel |
| 82336 | Repel |
| 6660 | Repel |
| 20745 | Inactive |
| 7802 | Repel |
| 7288 | Inactive |
| 76398 | Inactive |
| 77224 | Inactive |
| 10953 | Inactive |
| 14286 | Inactive |
| 60993 | Repel |
| 98218 | Inactive |
| 12665 | Inactive |
| 12753 | Inactive |
| 69326 | Inactive |
| 61265 | Inactive |
| 7410 | Inactive |
| 347476 | Repel |
