# Supplementary material for: Machine learning of honey bee olfactory behavior identifies repellent odorants in free-flying bees in the field
Source: eLife. 2026 Sep 23;14:RP104831. doi: 10.7554/eLife.104831 (PMC13600694; doi:10.7554/eLife.104831)
Supplement: Peer review history

# Editor report: eLife Assessment

**DOI:** 10.7554/eLife.104831.3.sa0

**Authors:** Claude Desplan

**Affiliations:** https://ror.org/0190ak572 New York University United States

**Keywords:** Important, Solid

This **important** manuscript reports a very interesting view of how pesticides can be toxic to beneficial insects like the honeybee. The study uses machine learning for the discovery of new honeybee-repellent odorants. The **solid** evidence predicts compounds that were validated in the lab and in the field. This work will be of great interest to researchers in ecology, pest control and sensory biology.

# Referee report: Reviewer #1 (Public review)

**DOI:** 10.7554/eLife.104831.3.sa1

[Editors' note: this version has been assessed by the Reviewing Editor without further input from the original reviewers. The authors have addressed the comments raised in the previous round of review.]

Original review:

Summary:

This manuscript reports a very interesting, novel and important research angle to add to the now enormous interest in how pesticides can be toxic to beneficial insects like the honey bee. Many studies have reported on how pesticides in standard use formulations show both lethality as well as sublethal negative effects on behavior and reproduction. The authors propose to use machine learning algorithms to identify new volatile compounds that can be tested for repellency. They use as input chemical structures that are derived from chemicals that have known repellent effects as identified in their initial behavioral assays.

Strengths:

The conclusion is that such chemicals specific to repelling bees and not pest insects (using the fruit fly as a model for the latter) can be identified using the ML approach. Have a list of such chemicals that can be rotated among in any field application would be a benefit because of the honey bees' ability to learn its way around any kind of stimulus designed to keep it from nectar and pollen, even when they may be tainted by pesticide.

Weaknesses:

The use of machine learning seems well-executed and legitimate. But this is beyond my expertise. So other reviewers can maybe comment more on that.

The behavioral data report on the use of a two-choice assay for bees in small Petrie plates. Bess can feed from two small wells place of filter paper impregnated with control or the control containing a chemical. The primary behavior, for ex in Fig 2C, is the first choice by one of the five bees in the plate of which well to feed from. For some chemical compound, there seems to be a 50:50 choice, indicating no repellent effects. In other cases the first bee making the choice chose the control, indicating possible repellent effects of the test chemical. Choices in this assay were validated in a free flying assay.

Concerns with the choice assay:

- 50-70 microliters amounts to what one hungry bee will drink. Did the first bee drink most of it, such that measures of bait consumed reflect a single bee or multiple bees?

- How many bees were repelled to the control side? Was it just the one bee? Were other measures considered? E.g. time to first approach; the number of bees feeding at different time points; the total number of bees observed feeding per unit time.

# Referee report: Reviewer #2 (Public review)

**DOI:** 10.7554/eLife.104831.3.sa2

Original review:

Summary:

The search for new repellent odors for honey bees has significant practical implications. The authors developed an iterative pipeline through machine learning to predict honey bee-repellent odors based on molecular structures. By screening a large number of candidate compounds, they identified a series of novel repellents. Behavioral tests were then conducted to validate the effectiveness of these repellents. Both the discovery and the methodological approach hold value for related fields.

Strengths:

* The study demonstrates that using molecular structures and a relatively small training dataset, the model could predict repellents with a reasonably high success rate. If the iterative approach works as described, it could benefit a wide range of olfaction-related fields.

* The effectiveness of the predicted repellents was validated through both laboratory and field behavioral tests.

Weaknesses:

The small size of the training dataset poses a common challenge for machine learning applications. However, the authors did not clearly explain how their iterative approach addresses this limitation in this study. Quantitative evidence demonstrating improvements achieved in the second round of training would strengthen their claims. For instance, details on whether the success rate of predictions or the identification of higher-affinity components would be helpful. Furthermore, given that only 15 new components were added for the second round of training, it is surprising that such a small dataset could result in significant improvements.

# Referee report: Reviewer #3 (Public review)

**DOI:** 10.7554/eLife.104831.3.sa3

Original summary:

The manuscript of Kowalewski et al. titled "Machine learning of honey bee olfactory behavior identifies repellent odorants in free flying bees in the field" did machine learning to predict potential candidates for honeybee repellents, which may keep foraging bees from pesticides. This is a pilot research with strong significance in the research of olfactory behavior and in pest control.

# Author comment: Author response

**DOI:** 10.7554/eLife.104831.3.sa4

**Authors:** Joel Kowalewski, Barbara F Baer-Imhoof, Tom Guda, Matthew Luy, Payton Arianna Rao, Boris Baer, Anandasankar Ray

**Affiliations:** University of California, Riverside Riverside United States; University of California Riverside Riverside United States; University of California, Riverside Riverside United States; University of California, Riverside Riverside United States; University of California, Riverside Riverside United States; University of California Riverside Riverside United States; University of California, Riverside Riverside United States

The following is the authors’ response to the original reviews.

**Public Reviews:**

**Reviewer #1 (Public review):**

Summary:

This manuscript reports a very interesting, novel and important research angle to add to the now enormous interest in how pesticides can be toxic to beneficial insects like the honey bee. Many studies have reported on how pesticides in standard use formulations show both lethality as well as sublethal negative effects on behavior and reproduction. The authors propose to use machine learning algorithms to identify new volatile compounds that can be tested for repellency. They use as input chemical structures that are derived from chemicals that have known repellent effects as identified in their initial behavioral assays.

Strengths:

The conclusion is that such chemicals specific to repelling bees and not pest insects (using the fruit fly as a model for the latter) can be identified using the ML approach. Have a list of such chemicals that can be rotated among in any field application would be a benefit because of the honey bees' ability to learn its way around any kind of stimulus designed to keep it from nectar and pollen, even when they may be tainted by pesticide.

Weaknesses:

The use of machine learning seems well-executed and legitimate. But this is beyond my expertise. So other reviewers can maybe comment more on that.

The behavioral data report on the use of a two-choice assay for bees in small Petrie plates. Bess can feed from two small wells place of filter paper impregnated with control or the control containing a chemical. The primary behavior, for ex in Fig 2C, is the first choice by one of the five bees in the plate of which well to feed from. For some chemical compound, there seems to be a 50:50 choice, indicating no repellent effects. In other cases the first bee making the choice chose the control, indicating possible repellent effects of the test chemical. Choices in this assay were validated in a free flying assay.

Concerns with the choice assay:

50-70 microliters amounts to what one hungry bee will drink. Did the first bee drink most of it, such that measures of bait consumed reflect a single bee or multiple bees?

The measure of lure consumed reflects multiple bees. We observed that the first bee did not empty the 70 ul of honey, allowing us to estimate honey consumption by several bees.

How many bees were repelled to the control side? Was it just the one bee?

All the bees in a group were repelled to the control side for repellents. Evaluating lack of honey consumption, also allowed us to repellency as well. As an example: if 100% honey is consumed on the control side meant that the bees were hungry, but if 0% honey was consumed on the repellent side, this meant that the bees were not hungry enough to drink from the honey on the repellent side.

Were other measures considered? E.g. time to first approach; the number of bees feeding at different time points; the total number of bees observed feeding per unit time.

Bees were cooled down to place them in the plates for the experiments. Therefore, time to first approach could also depend on how long it took the bees to warm up, which was not as relevant for our research question. Because bees can communicate where to find food sources to each other, we restricted ourselves to first choice, only, to get independent data points for each plate. However, we investigated whether the first cup the first bee chose was also the one it drank from, which was the case.

**Reviewer #2 (Public review):**

Summary:

The search for new repellent odors for honey bees has significant practical implications. The authors developed an iterative pipeline through machine learning to predict honey bee-repellent odors based on molecular structures. By screening a large number of candidate compounds, they identified a series of novel repellents. Behavioral tests were then conducted to validate the effectiveness of these repellents. Both the discovery and the methodological approach hold value for related fields.

Strengths:

The study demonstrates that using molecular structures and a relatively small training dataset, the model could predict repellents with a reasonably high success rate. If the iterative approach works as described, it could benefit a wide range of olfaction-related fields.

The effectiveness of the predicted repellents was validated through both laboratory and field behavioral tests.

Weaknesses:

The small size of the training dataset poses a common challenge for machine learning applications. However, the authors did not clearly explain how their iterative approach addresses this limitation in this study. Quantitative evidence demonstrating improvements achieved in the second round of training would strengthen their claims. For instance, details on whether the success rate of predictions or the identification of higher-affinity components would be helpful. Furthermore, given that only 15 new components were added for the second round of training, it is surprising that such a small dataset could result in significant improvements.

The original repellency dataset was collected from multiple older studies, each with differences in assays for bee behavior, and using differing delivery and chemical concentrations. Moreover, the number of strong repellents were limited in number, and because they varied structurally from non-repellents in the dataset, the AUC appeared high. A smaller dataset result in unusual AI/ML model performance trends, as any algorithm is just a reflection of its training data. As a result, we found that the Round 1 predictions had a low success rate in behavior assays (~20%). Subsequently, even small amounts of data collected using one standard concentration and assay, could dramatically change the quality of the dataset, not just for structures of repellents, but also related structures that were not repellent. What we observe is a more complete representation of how repellents and non-repellents are distributed when adding just 15 chemicals. And the prediction success of Round 2 is more than doubled in repellent behavior assays at >50%. The initially observed performance gains with even small additions to the training dataset will stabilize and ultimately plateau due to the limits of the ML algorithm and/or chemical featurization technique. A more complex model, trained on a large dataset, may not be expected to benefit from a handful of additional examples, it is because the chemical feature distributions are already better approximations of the real world. To put simply, smaller datasets imply there is more to learn.

It is also true that the size of the training dataset is important for AI/ML algorithms, Artificial neural network, for instance, are highly sensitive to noise and generalize poorly with limited data; the noise is amplified in these cases, and the solution—reducing the complexity of the model—impedes learning. Many algorithms like the decision trees and support vector machines featured in our paper can handle noise more efficiently and are suitable for smaller datasets in that they can still make reasonably successful predictions.

**Reviewer #3 (Public review):**

The manuscript of Kowalewski et al. titled "Machine learning of honey bee olfactory behavior identifies repellent odorants in free flying bees in the field" did machine learning to predict potential candidates for honeybee repellents, which may keep foraging bees from pesticides. This is a pilot research with strong significance in the research of olfactory behavior and in pest control. However, some major issues need to be addressed to enhance the manuscript's clarity, strength, and overall coherence.

(1) *Drosophila melanogaster* is not considered as a true agricultural pest. The manuscript would be more compelling if using true pests, for example, *Drosophila* suzukii or others.

Honeybees face a critical risk of lethal pesticide exposure when they drift from their designated orchards into adjacent blooming crops or honeydew-coated fields, where they encounter chemical treatments intended for insects like Citrus Thrips, Asian Citrus Psyllid, Alfalfa Weevil, Peach Twig Borer, Oriental Fruit Moth, Lygus Bugs , Cotton Aphids, Whiteflies, Corn Rootworm, Sunflower Head Moth, Vine Mealybug, Cucumber Beetles, and Sugarcane Aphids. Unfortunately, testing such pest species is outside the scope of this paper, but would deserve further research.

(2) For repellency test, the result relies on dosage. An attractant may become a repellent at high concentration. Test a range of concentrations for each chemicals and compare responses between honeybees and pests.

Testing freely flying honey bees in the field is an extremely challenging undertaking. Nevertheless, we added extra tests for two strong repellents, BR4.5 and BR3.81, at half dose of 0.05 mg/cm^2^. As expected, we found that there was a reduction in repellency. Testing more concentrations was not within the scope of this paper.

(3) Be more clear about bee behavior data and their scores (as in Page 4 Results "184 training chemicals and later for 203 chemicals" and Page 10 Methods). I suggest that authors add a supplemental table with each chemical and its behavioral score, feature and reference - which ones were used for training, and which ones for testing. Also add your own behavioral test data (second input) to this table

We have added the training chemical lists as Supplemental Tables S3 and S4.

(4) The AUC in the first validation was 0.88 (Page 4), and in Page 5, "As expected, the computational validation results based on the AUC values, show an improvement." However, there were no other AUC values to show improvement.

(5) Show plots of ROC AUC curves from Round 1 and Round 2.

The round one ROC curve is shown in Figure 1. The round two ROC curves obtained from 3 different approaches (Author response image 1). The manuscript shows direct behavioral validation of chemicals identified, which is more important.

**Author response image 1.**

(6) In the Discussion, the authors mentioned olfactory receptors in honeybees. It would be useful to provide a general review of the current understanding of these receptors and their (potential) functions.

We have expanded the discussion and pointed to a review on honey bee olfaction.

(7) I suggest combining Fig. 1 and Fig. 3A as one pipeline for this work.

(8) Figure 2C, some sample sizes are very small, such as 2-piperidone: 1 first-choice control vs 0 first-choice repellent? Increase sample size and do statistical analysis.

Most compounds except the one pointed out, have small sample sizes because of the low percentage of bees participating in the trials. Consequently, we improved methods in round 2 and were able to increase participation from 68% to 81%, as described in the methods. However since the compound was included in the second round of training, we would like to report it anyway. This compound had the highest rate of non-participating plates compared to the others and there is a possibility that it it may affect both the stimuli.

(9) In general, to assist reviewers, include line numbers to the manuscript.

**Recommendations for the authors:**

**Reviewer #1 (Recommendations for the authors):**

Other factors about the newly identified chemicals:

Is there a toxicity index for these chemicals that can be listed? This would be important obviously for any humans around the repellents

While toxicity index determination is outside the scope of this manuscript, it is possible to predict Rat LD50 values using the EPA Suite’s toxicity prediction tool. In a pilot test, the software predicted an average oral toxicity is ~3064mg/kg for the 18 repellents in Round 2, which is considered “Practically non-toxic” by the EPA.

Was there any indication of bees being behaviorally impaired or dying when exposed to the chemicals in a confined space? Even exposure to intense floral perfumes in a confined space and be toxic over a longer period.

Less than 5% of the 2225 honey bee died after the experiments, and none of the compounds showed a significantly higher level of dying, suggesting that the minor effect was not due to chemicals, but possibly due to handling steps (starving, chilling, recovery, etc).

The 'plates not participating' measure indicates plates in which no bees fed on either choice. Is that correlated to the choice index? That is, when bees showed some repellency was it the case that often that led to no choice?

Yes, non-participating plates were those, in which the bees did not drink any honey at all. The reason for this could have been that the bees were too cold and unable to heat up enough to participate in the trials, or that the chemical was so repellent, the bees did not want to drink any honey at all. Because we were not able to distinguish between these two reasons, we excluded plates in which the bees did not drink any honey at all from our dataset.

It is unclear why the McNemar test was used.

The McNemar test is used for hypothesis testing for paired dichotomous data. In our data file, we created two columns to report our first-choice results: “Control side first” and “Repellent side first”. When the first bee in a plate drank from the control side first, we added a 1 to the “Control side first” column and a “0” to the “Repellent side first” column. Because one control and one repellent-side honey pot were in the same Petri dish, the bees could only choose one side first, this meant it could not choose the other side at the same time. Consequently, our dataset consisted of paired samples, which were dependent from each other. We therefore split the dataset by Repellent candidate, and we used the paired -sample McNemar tests for non-parametric data. (Lachenbruch P.A. McNemar Test, Wiley StatsRef: Statistics Reference Online)

The statistical result is not discussed in the text, only shown in the figure. And it looks to be significant only for one chemical and DEET. Yet on page 4 the end of the second paragraph, the authors write "For many of the tested compounds the bees preferred to visit the honey-water pots on the control side versus the repellent side,". That implies that they are not really using the test as a meaningful means for showing differences. If they are arguing only from trends, then that should be clearer in the text.

We reported the p-values for each test we had used in tables in Figure 2C and S2. In the methods section we report which statistical tests were used to evaluate the data.

There is no mention of attractant chemicals:

Slessor and Winston used queen pheromone to attract bees to fields and improve pollination. Honey bees use the Nasonov pheromone to attract other bees to feeding locations. Could the addition of their chemical features change ML outcomes? This should be at least discussed.

We thank the referee for the suggestion; however the focus this manuscript is repellents and therefore we restricted the background to that area of knowledge.

**Reviewer #2 (Recommendations for the authors):**

Minor comments:

Releasing the dataset and code will benefit the readers interested in this study.

The behavioral data are reported within the figures, tables, and supplementary. The computational code will be available upon request from the communicating author for non-commercial use.

Figure 1, AUC curve, "AUC = 0.XX", should there be an actual value from the experiment?

Added

Page 4, "(Talbe S1)" should be placed in the next sentence, as "From the initial training set we identified 45 features that were considered important for predicting aversive valence (Table S1)."

We have added this in the appropriate spot.

Page 5, "As expected, the computational validation results based on the AUC values, show an improvement.". Please list the AUC values.

**Author response image 2.**

**Reviewer #3 (Recommendations for the authors):**

Minor comments:

(1) Page 3: "they sense using a sophisticated olfactory system of >180 odorant receptor genes in the genome". In the cited Robertson & Wanner's paper, there are around 160 receptors, and 170 if pseudogenes are included.

We thank the referee and have updated the numbers.

(2) Page 4: "initially for 184 training chemicals and later for 203 chemicals (Table S1)." Table S1 is about features, not chemicals?

We have moved the reference to an appropriate location.

(3) Figure 2A: What is the control? Acetone or another solvent?

Acetone, but it rapidly evaporates before the time of experiment.

(4) Figure 2A: What does asterisks mean?

Statistically significant.

(5) Figure 3: When you added your own testing data as a second input for Round 2, put details about these data: chemical names, preference scores... Also, are Round 2 data (Round 1 plus your own) were also split as 90:10 into training and testing partitions?

Yes, the validation was performed on the updated data set including the new chemicals.

(6) Figure 3D: Is asterisk at correct location? What does it mean?

Means that BR3.15 was significantly different from BR4.5

(7) Figure 4D: "4D" in legend is missing. Also, "... tested at the regular dose (0.1mg/cm2) and half dose (0.05mg/cm2)". In the panel, it is only 0.05mg/cm2.

Added

(8) Table S2 is the same as Fig. 2C? Remove one.

We have deleted Table S2.
